# Detection, quantification and genotyping of Herpes Simplex Virus in cervicovaginal secretions by real-time PCR: a cross sectional survey

**DOI:** 10.1186/1743-422X-2-61

**Published:** 2005-08-11

**Authors:** Esther AN Aryee, Robin L Bailey, Angels Natividad-Sancho, Steve Kaye, Martin J Holland

**Affiliations:** 1 Medical Research Council Laboratories, Fajara, The Gambia; 2 London School of Hygiene and Tropical Medicine, London, UK

## Abstract

**Background:**

Herpes Simplex Virus (HSV) Genital Ulcer Disease (GUD) is an important public health problem, whose interaction with HIV results in mutually enhancing epidemics. Conventional methods for detecting HSV tend to be slow and insensitive. We designed a rapid PCR-based assay to quantify and type HSV in cervicovaginal lavage (CVL) fluid of subjects attending a Genito-Urinary Medicine (GUM) clinic. Vaginal swabs, CVL fluid and venous blood were collected. Quantitative detection of HSV was conducted using real time PCR with HSV specific primers and SYBR Green I. Fluorogenic TaqMan Minor Groove Binder (MGB) probes designed around a single base mismatch in the HSV DNA polymerase I gene were used to type HSV in a separate reaction. The Kalon test was used to detect anti-HSV-2 IgG antibodies in serum. Testing for HIV, other Sexually Transmitted Infections (STI) and related infections was based on standard clinical and laboratory methods.

**Results:**

Seventy consecutive GUM clinic attendees were studied. Twenty-seven subjects (39%) had detectable HSV DNA in CVL fluid; HSV-2 alone was detected in 19 (70%) subjects, HSV-1 alone was detected in 4 (15%) subjects and both HSV types were detected in 4 (15%) subjects. Eleven out of 27 subjects (41%) with anti-HSV-2 IgG had detectable HSV-2 DNA in CVL fluid. Seven subjects (10%) were HIV-positive. Three of seven (43%) HIV-infected subjects and two of five subjects with GUD (40%) were secreting HSV-2. None of the subjects in whom HSV-1 was detected had GUD.

**Conclusion:**

Quantitative real-time PCR and Taqman MGB probes specific for HSV-1 or -2 were used to develop an assay for quantification and typing of HSV. The majority of subjects in which HSV was detected had low levels of CVL fluid HSV, with no detectable HSV-2 antibodies and were asymptomatic.

## Background

Genital herpes, which is caused mainly by Herpes Simplex Virus (HSV) -2 [1] but also by HSV-1 [2] remains a worldwide problem [3]. The strongest known risk factor for the heterosexual transmission of Human Immunodeficiency Virus (HIV) and other Sexually Transmitted Infections (STI) is Genital Ulcer Disease (GUD) [4]. Over the past decade, HSV-2 has been identified as the most common aetiological agent of GUD [5]. Studies of HSV-2 seroprevalence have found high rates in African-Americans [6] and in African populations in Uganda, Zimbabwe, Tanzania, Central African Republic, South Africa and The Gambia [7-12]. In The Gambia, HSV-2 seropositivity among young adults from rural communities was 28% in women and 5% in men which increased with age [12]. HSV-2 seroprevalence increases with high risk sexual behaviour [9] and with factors related to polygynous marriage practices in rural populations [13]. The majority of subjects infected with HSV-2 are asymptomatic but exhibit sub-clinical cervicovaginal virus secretion which is thought to be important in the transmission of HSV-2 [14,15].

Antiviral therapy with acyclovir or valacyclovir, used during episodes of primary and recurrent HSV-2 GUD, reduces both the rate of secretion, and the rate at which GUD develops [16,17]. These drugs were also found to reduce the transmission from an infected person to another susceptible individual [18] and to minimise sub-clinical HSV-2 genital secretion, preventing the spread of disease [19]. Resistance to antiviral drugs has been reported but occurs infrequently [20]. Interventions therefore can be helpful in the reduction of disease by preventing the spread of HSV, which consequently impacts on HIV transmission. The effectiveness of such interventions demands rapid, efficient, reliable and type specific assays. These assays can serve as biological endpoints in deciding when to administer intervention, monitoring the effectiveness of any current intervention, determining the efficacy of drugs, assessing drug resistance and are useful research tools in the study of the epidemiology of transmission.

The discrimination of HSV-1 from HSV-2 was originally performed using virus culture followed by antibody binding to type-specific determinants (virus neutralisation) [21]. The application of molecular methods, such as restriction fragment length polymorphism (RFLP) analysis of HSV PCR amplicons is thought to provide a reliable method of typing the virus [21]. Whilst serology based typing methods target surface exposed epitopes such as those on glycoprotein C or G, molecular typing has largely exploited differences between HSV-1 and -2 DNA polymerase I genes. Archetypal HSV-1 and -2 DNA polymerase I genes share 93% sequence identity and 82% amino acid homology. The selection of strain typing polymorphisms for molecular methods is based on the sequence information deposited in public databases coupled with the availability of a convenient restriction endonuclease site. This can identify variation at a selected single nucleotide polymorphism (SNP) site. A rapid SNP typing method is useful because it can yield information about the virus population in the affected host population. This is of value in classification and in epidemiological studies aimed to investigate host-pathogen interplay.

A more efficient method for diagnosis of HSV infection is to use PCR in real time for detection and quantification. HSV SNP sub-typing by 'allele' specific fluorogenic probes offers many advantages over RFLP methods or viral culture. Amplification of the target DNA, and hybridization to a fluorogenic probe are conducted in a single PCR and therefore the chances of possible contamination are minimised. The main advantage of real-time detection is the large dynamic range offered in a quantitative assay coupled to the ability to discriminate between fluorophores in a multiplex reaction. We selected Taqman probes incorporating the minor groove binder (MGB), 1,2-dihydro-(3*H*)-pyrrolo [3,2-*e*] indole-7-carboxylate (CDPI_3_) [22]. MGB probes offer high sensitivity and accuracy, due to their short length which increases the sensitivity and stability of probe-sequence complexes to single base changes [23]. However, important consideration should be given to the selection of the SNP under investigation. Recent work using Eclipse-MGB probes which bind to a highly polymorphic region of HSV glycoprotein D found that sequence polymorphisms in the probe binding region decreased the sensitivity of typing assay [24]. The present study used assays based on Taqman-MGB probes, to identify the HSV type in a population of symptomatic and asymptomatic patients attending a GUM clinic in The Gambia. The possible role of other co-infections in the secretion of HSV in CVL fluid and HSV transmission were investigated.

## Results

### Study subjects

Seventy subjects included in the study were of median age 27 years (range 17–50). Genital examination revealed that 5/70 subjects had GUD (four external and one cervical). Four subjects withheld consent for HIV serology. There was one known HIV positive patient identified at a previous Out Patient Department visit. Two further samples were not tested for HSV-2 IgG (total tested n = 63) and one sample was not tested for Hepatitis B and *Treponema pallidium *(n = 62) because of insufficient sample volume.

### Quantitative analysis of HSV viral load in CVL

**Figure 1:**
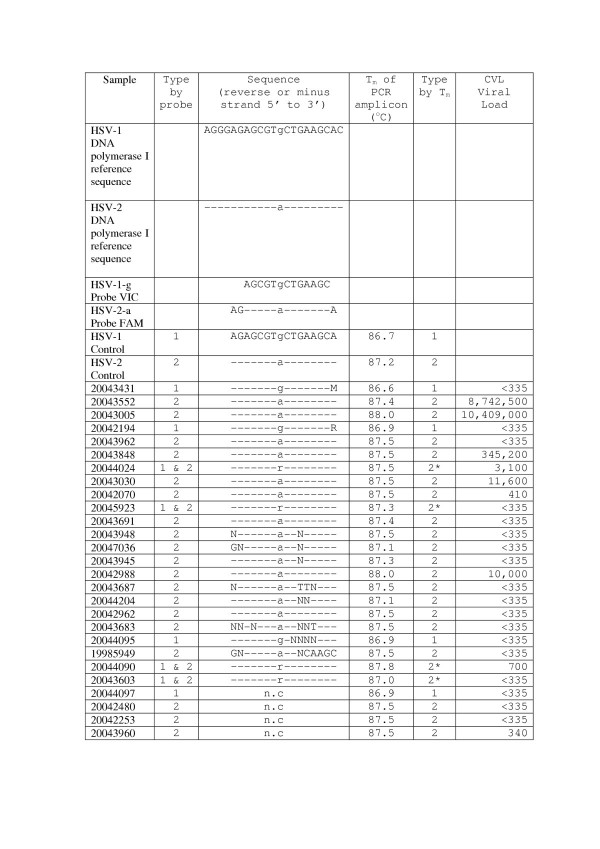
R = A/G; M = A/C; N = A/T/G/C. Control HSV-1 and -2 DNA was obtained from 2003 Quality Control Molecular Diagnostics 2003 Proficiency panel (Block 6, Kelvin Campus, West of Scotland Science Park, Glasgow UK). The reference sequences were based on Blast results from NCBI. The T_m _of the PCR product (146 b.p.) which was amplified during quantitation with SYBR Green I is shown against its complementary sequence with the SNP position marked in **bold**. The probe and sequence ascertainment of types were in agreement. * T_m _was unable to distinguish dual infection in this assay. -n.c. = not confirmed by sequencing.

Amplification of the HSV DNA and hybridization to a fluorogenic probe were conducted in different PCR reactions. Three μl of extracted template DNA of a 200 μl eluate prepared from CVL was used. Most samples had values less than 10 viral copies/PCR reaction but were positive as indicated by the presence of an amplification peak of the correct T_m _in dissociation or melting curve analysis plots. The accurate estimation of the quantity of HSV was expressed as viral copies/ml of CVL. The lower limit of quantitation of the HSV assay was therefore 335 viral copies/ml cervical lavage fluid. Forty-three samples had no detectable HSV amplicons. Twenty-seven out of 70 subjects (39%) had HSV detected in CVL fluid. Figure 1. shows the HSV load in the CVL fluid of these 27 HSV PCR positive subjects. The distribution is left censored and the majority of subjects had <335 copies/ml of lavage. HSV secretion by quantitative real time PCR in positive subjects ranged from < 335 to 10,409,000 viral genome copies/ml of CVL fluid.

### Determination of HSV-1 and -2 types by Taqman-MGB probes

Nineteen of the 27 HSV positive subjects (70%) were identified as HSV-2, 4/27 (15%) were HSV-1 and 4/27 (15%) were positive for both HSV-1 and -2 by HSV probe specific binding assay. HSV type was confirmed in 7 samples in which an equivocal typing result was initially obtained. HSV type was confirmed by repeating the real-time assay using the Rotorgene 3000 instrument (Corbett Research Ltd, Sydney, Australia). Sequencing of all PCR amplicons and reference control strains gave a 100% confirmation with that of the probe at the SNP site (Figure 1).

### HSV type and serology

Anti HSV-2 IgG antibodies were detected in 29/63 (46%) subjects, most of whom were not secreting HSV. The subject with the highest number of HSV-2 viral genome copies/ml of CVL fluid had no detectable anti HSV-2 IgG. Two other CVL fluid samples in which high levels of HSV DNA were detected were typed as HSV-2 and these were positive for anti HSV-2 IgG antibodies. In total 10/21 subjects in whom HSV-2 secretion was detected were negative for HSV-2 antibodies, whilst 11/21 subjects in which HSV-2 secretion was detected were HSV-2 antibody positive. Two subjects in which HSV-2 secretion was detected were unable to be tested for anti HSV-2 IgG due to insufficient serum. A single HSV-1 secretion positive subject was positive for anti HSV-2 IgG and 3 HSV-1 secretion positive samples were negative for anti HSV-2 IgG antibodies.

### Potential risk factors which may be associated with HSV cervicovaginal secretion

Possible cofactors for genital HSV infection were examined (Table 1) but none of these were associated with current HSV secretion in CVL fluid. Data in Table 2 further demonstrates the lack of a relationship between each risk co-factor and the quantity of HSV present in CVL. Eighteen (67%) subjects judged to be secreting HSV had < 335 viral copies / ml of CVL fluid. Five of 70 subjects had GUD, 2 of 5 were currently secreting HSV (HSV-2 by typing and positive for anti-HSV-2 IgG). Seven of 66 subjects (11%) were found to be HIV-1 seropositive. Thirty-five out of 70 (50%) were diagnosed as having Bacterial Vaginosis (BV). Twenty-nine of 70 (41%) subjects had *Candida *and *Trichomonas vaginalis *was found in 7 out of 70 (10%). Eight out of 62 (13%) were positive for Hepatitis B and 7 out of 70 (10%) were positive for *Chlamydia trachomatis *pgp3. None of the subjects were diagnosed as having *Neisseria gonorrhoea*, or clue cells. Two of 62 (3%) subjects were diagnosed as *T. pallidium *infected, neither of which secreted HSV.

**Table 1:** Association between potential risk factors and HSV detected in CVL

**Cofactors**	Present	Absent	Relative risk **(95% CI)**	P-value
GUD	2/5 (40%)	25/65 (38%)	1.04 (0.34 – 3.18)	0.68
Anti HSV-2 IgG* (Kalon test)	11/27 (41%)	7/32 (22%)	1.86 (0.84 – 4.13)	0.19
HIV	3/7 (43%)	23/59 (39%)	1.1 (0.44 – 2.74)	0.83
BV	14/35 (40%)	13/35 (37%)	1.08 (0.60 – 1.95)	1.00
*C. trachomatis*	5/7 (71%)	22/63 (35%)	2.05 (1.15 – 3.64)	0.14
Hepatitis B	5/8 (63%)	22/54 (41%)	1.53 (0.82 – 2.87)	0.44
*Candida*	9/29 (31%)	18/41 (44%)	0.71 (0.37 – 1.35)	0.40
*T. vaginalis*	3/7 (43%)	24/63 (38%)	1.13 (0.45 – 2.80)	0.86

* Comparison with HSV-2 only. P values calculated by χ^2 ^test

**Table 2:** Relationship of HSV CVL viral load with potential risk factors

Cofactor	Geometric mean number of copies of virus/ml of lavage fluid		
	
	Present	Absent	P-values
GUD	8900	<335	0.1532
Anti HSV-2 IgG	1100*	<335*	0.2976
HIV-1 infection	<335	<335	0.5568
BV	400	<335	0.5020
*Candida*	<335	<335	0.8320
Hepatitis B	400	<335	0.2480
*C. trachomatis*	<335	<335	0.1890
*T. vaginalis*	17100	<335	0.0760

*Comparison done with HSV-2 only. P values calculated by non-parametric Kruskal-Wallis test.

### Determination of HSV-1 and -2 types by T_m_

**Figure 2:**
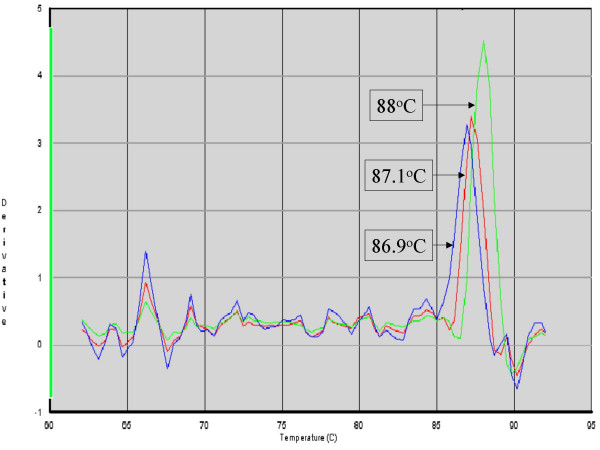
Dissociation curves of amplicons used to identify HSV-1, HSV-2 and dual positive samples. No curves were observed in HSV negative subjects. T_m _was recorded and compared with probe binding and sequence results. Collectively nine different peaks with T_m _in the range 86.6 – 88.0°C could be observed. Three T_m _representative of HSV-1, HSV-2 and dual positive samples confirmed by sequencing are shown with T_m _indicated.

The melting temperatures (T_m_) of the HSV amplicon obtained from the dissociation or melting curve plot following amplification and quantification of HSV are shown in Figure 1. Both positive and control HSV-1 samples had T_m _ranging from 86.6°C to 86.9°C. HSV-2 positive samples, ranged from 87.0 – 88.0 °C and an example of the dissociation plot is shown in Figure 2. Dual HSV -1 and -2 infections, confirmed by sequencing, also had a T_m _range between 87.0–88.0°C. Of note in the sequences is the high number of mutations or alternative bases contained with in the probe binding area. Surprisingly probe binding and T_m _appear largely unaffected by these changes (Figure 1). Of the 7 samples confirmed by Rotorgene, 4 of these had no mutations in the probe binding area. The remaining 3 samples resulted in poor quality sequencing reactions and the results were not interpretable.

## Discussion

Diagnostic methods, ranging from traditional culture and serological detection methods, to molecular techniques, have been described for the diagnosis of HSV. Most of these assays, including the gold standard of viral isolation by culture [25] are slow and prone to contamination. The assay turn-around time for culture is 4 days as compared to that of 4 hours for enzyme immunoassay (EIA) and 2–4 hours for real-time PCR [26]. Viral culture diagnosis is useful if HSV-2 is responsible for symptomatic infection in the form of vesicles or ulcers, when live virus can usually be isolated. Success of detection further depends on the secretion of virus during sampling. Its sensitivity relies on the way samples are collected, transported and stored [21]. Cell culture can only be done in laboratories with expertise and facilities; in developing countries this facility may not be available. Accurate serological tests are appropriate in asymptomatic cases, when viral culture and PCR assays are largely negative [15].

Several commercially available HSV-type specific serological assays are available, but a test such as a HSV-2 Western blot is expensive and restricted largely to reference laboratories. The Kalon test, which was found to be the best among a set of serological tests evaluated in samples from different African cities (with sensitivity and specificity of 92.3% and 97.7% respectively) [27], tests only latent infection and may not detect recent seroconversion [28]. DNA amplification using PCR techniques is reported to be more sensitive than culture, and a number of studies have used fluorescent based real time PCR techniques with primers targeting sequences from HSV glycoprotein B, thymidine kinase or DNA polymerase genes [21,29]. Some PCR assays require laborious post-PCR procedures such as RFLP analysis [21], which may introduce a risk of contamination. The high degree of sequence homology between HSV-1 and -2 makes the design of type specific primers challenging [30], nevertheless this has been attempted with varying success, along with Amplification Refractory Mutation System PCR [31].

Our assays were able to estimate HSV load and distinguished specific HSV-1 and -2 cervicovaginal viral secretion. Amplification and typing could not be carried out in a single PCR because, under the conditions used, insufficient specific amplicons were generated for accurate typing in a single step. This may have circumvented problems relating to sensitivity of the HSV-1 and -2 probe relative binding. In a single step multiplex assay mutations in the probe binding area are reported to lead to a loss in sensitivity and error in the classification of samples [24]. When diluted amplified HSV amplicons were used for a further typing reaction, HSV-2 was more commonly detected than HSV-1 (70% opposed to 15%). This is concordant with recent work that found most subjects were secreting HSV-2 [32]. An earlier study of Gambian commercial sex workers found that 26% of women were secreting HSV but the study could not distinguish HSV strain types (Aryee *et al *unpublished observation). In the current study most of the subjects secreting HSV were of age ranging 20 – 41 years. This confirms earlier studies in which HSV-2 was most prevalent (15 – 34 year old subjects from rural Gambian communities) [12]. Thus Gambian women in their twenties appear at highest risk of HSV-2 infection. Most of the women reported in this study were found to be secreting low levels of viral DNA in CVL fluid. Anti HSV-2 IgG was detected in 29 out of 63 (46%) subjects which is higher than a previous Gambian study by Shaw *et al *[12]. The subjects for that study were from rural Gambian communities whereas our work was with GUM clinic attendees, whose risk of HSV-2 infection is greater than the general population [33]. HIV has been found to enhance the expression of HSV-2 [11]. However, it is unlikely that co-infection with HIV is responsible for the increased HSV-2 sero-prevalence in this study given the low rate of HIV infection among the study subjects and the low level of HSV secretion in HIV-1 positive subjects compared to HIV-1 negative subjects.

We found anti HSV-2 IgG seropositivity correlated poorly with HSV-2 secretion and several factors may have contributed to this observation. IgG seropositivity may take time to develop and the Kalon antibody test may not be sensitive enough to detect early seroconversion. HSV-2 could therefore be present in secretions without established sero-conversion. It is known that HSV-2 genital secretion is intermittent even in HSV seropositive subjects so it is possible that these subjects have only recently been exposed such that a detectable immune response has not yet developed. The highest HSV-2 viral load in lavage fluid was found in a seronegative subject. This result may not be conflicting if this was a newly acquired infection and only a primary antibody (IgM) response was stimulated with levels of IgG below the sensitivity of the Kalon test. Follow-up of subjects is required to investigate whether subjects in which HSV secretion was identified but were seronegative for anti HSV-2 IgG have now seroconverted.

The data suggest that while STI such as BV, HIV, *C. trachomatis *and Hepatitis B may increase the rate and quantity of HSV-2 secretion, these effects were not statistically significant which is probably due to the low numbers of women recruited for the study. The study suggested that subjects with GUD tend to secrete more virus than those without GUD, however, most subjects that were secreting virus were asymptomatic and did not have GUD in line with earlier work [16,17]. The melting temperature (T_m_) of PCR amplicons has been used to identify HSV types by others [24,32] but these methods could not distinguish between dual and mono specific HSV-2 infection. The use of amplicon T_m _for the assignment of a genotype has been utilized for human SNPs using High-Resolution melting instruments [34]. Whilst this may be applicable for the relatively stable sequences in the human genome it may not be a sustainable method for highly changeable viral sequences as suggested by others.

The T to C (A/G in the reverse strand) transition that we have identified as a distinguishing SNP appears to be indicative of either HSV-1 or HSV-2, however, there are a limited number of HSV sequences available in public sequence databases to indicate that every HSV-1 or 2 will have either T or C at that position. It has not been demonstrated that these SNPs, PCR-RFLPs or T_m _correlate with monoclonal type specific antibody reactivity or unique region sequence data. Further data need to be gathered to evaluate the usefulness of these methods and their application to population and epidemiological studies.

## Conclusion

This assay was able to distinguish HSV-1 from HSV-2 and quantify HSV genital secretion. Thirty nine percent of women attending the GUM clinic were secreting HSV and most of these had low viral loads in CVL fluid with no detectable anti-HSV-2 IgG antibodies and were asymptomatic. The presence of other STI may facilitate HSV secretion but further studies with a larger sample size are required to investigate whether the HSV type or whether low levels of HSV genital secretion are important in the transmission of infection.

## Methods

### Subjects

Seventy consecutive female subjects, attending the GUM clinic at MRC Fajara, The Gambia from April to June 2004 were recruited. After giving informed consent, clinical data about the subjects were recorded. This was conducted by questionnaire and an examination for genital lesion by the clinician/nursing officer. The study was approved and conducted under the guidelines of The Gambian Government and MRC Joint Ethics Committee.

### Specimens

Two vaginal/cervical swab specimens were taken, after which the cervicovaginal area was flushed with 10 ml of phosphate buffered saline (PBS) for 1 minute and aspirated into sterile tubes. Samples were kept on ice and transported promptly to the laboratory. One ml of venous blood was also collected and allowed to clot before centrifugation at 800 × *g *for 10 minutes to isolate serum. Serum was stored at -20°C until used.

### Processing specimens

Cervicovaginal lavage samples were centrifuged at 1000 × *g *for 10 min and the supernatant discarded. Cellular materials were resuspended in 1 ml PBS and stored at -70°C. A high vaginal swab was used to make a smear on clean slides for Gram staining. The second swab was used for routine microbiological analysis of STI.

### DNA extraction

DNA was extracted from 200 μl of lavage cell suspension using the QiaAmp DNA Mini kit (QIAGEN Ltd, Crawley, UK) according to manufacturers instructions.

### Selection of HSV typing single nucleotide polymorphism

A survey of HSV-1 and -2 DNA polymerase I gene sequences available through the National Center for Biotechnology Information (NCBI)  was conducted. Eight HSV-1 [EMBL:X03181.1], [EMBL:X04495.1], [EMBL:X04771.1], [EMBL:X14112.1], [DDBJ:AB072389.1], [DDBJ:AB070848.2], [DDBJ:AB070847.2], [GenBank:M10792.1] and 5 HSV-2 [GenBank:AY038367.1], [EMBL:Z86099.2], [GenBank:M14793.1], [GenBank:M16321.1], [GenBank:AY038366.1] sequences were identified. Following alignment, candidate SNPs were selected in regions with no other base changes within 20 nucleotides (*i.e. *within the likely probe binding area) of the potential typing SNP. These SNPs were then submitted for primer-probe design using either Primer Express v2.0 (Applied Biosystems Inc, Warrington, UK) or using the web based service of Epoch Biosciences . The optimum primer-probe design combination was then selected for synthesis.

### Detection of HSV DNA, probe typing & melting point determination

Quantitative PCR was performed on the ABI 5700 sequence detection system (Applied Biosystems Inc, Warrington, UK). Duplicate 3 μl samples of extracted DNA were added to a 22 μl PCR master mix (PCR SYBR Green I, QIAGEN Ltd, Crawley, UK) containing 0.4 μM each primer. The primers amplified a generic HSV 146 b.p. product from the HSV DNA polymerase I gene. [forward primer – 5'-AGCCTGTACCCCAGCATCAT-3'; reverse primer – 5'-TGGGCCTTCACGAAGAACA-3']. Cycling temperatures were 95°C for 15 minutes, followed by 40 cycles of 94°C for 15s, 58°C for 30s and 72°C for 30s. At the end of amplification PCR products were subjected to a dissociation or melting curve analysis and the T_m _of the peak was recorded. HSV PCR products were diluted one in ten with DNase free RNase free water and probe typed using Taqman MGB probes (Applied Biosystems, Inc, Warrington, UK) directed against the HSV DNA polymerase I gene. These probes specifically detected a C/T SNP at position 2202 (C) for HSV-1, and 2451 (T) for HSV-2. The probe sequences were: HSV-1 (5'-VIC-AGCGT**g**CTGAAGC-MGB-Q-3') and HSV-2 (5'-6FAM-AGAGCGT**a**CTGAAGCA-MGB-Q-3'). The probes were used in a single tube real time PCR using the QuantiTect probe kit (QIAGEN Ltd, Crawley, UK) with the following cycling conditions using either an Opticon 2 (GRI/MJ Research, Braintree, UK) or Rotorgene 3000 (Corbett Research, Sydney, Australia) thermal cycler: 95°C for 15 minutes, followed by 40 cycles of 94°C for 15s, 68°C for 30s and 76°C for 30s. Fluorescence was acquired at the end of the annealing phase. For the Rotorgene 3000 HSV typed samples, an annealing phase of 66°C for 30s was used. The quantitative assays performed on the ABI sequence detection system included standards of 10^6 ^to 10 copies per reaction and negative controls. These were used to generate a standard curve and calculate the copy number of the unknown samples. HSV positive samples from the Quality Control Molecular Diagnostics 2003 Proficiency Panel (Block 6, Kelvin Campus, West of Scotland Science Park, Glasgow UK) were used as positive controls. All DNA samples were tested for inhibition of PCR using bacteriophage lambda (λ) DNA and primers. Briefly, test samples were 'spiked' into a PCR reaction containing approximately 100 copies of bacteriophage λ DNA and a primer pair directed against λ DNA. The performance of the PCR was monitored by quantitative real-time PCR (qPCR). The mean cycle threshold (Ct) and the standard deviation of the controls were calculated. Samples in which the mean Ct of the test sample fell outside the mean Ct plus three standard deviations of the controls, were judged to be inhibitory. Inhibitory samples were re-extracted by a repeat of the QiaAmp Mini kit extraction method and retested in the qPCR and inhibition assays.

### HSV amplicon sequence confirmation of probe typing

One in ten dilutions of the amplified positive products were prepared using DNase free RNase free water. A PCR reaction was prepared by adding 6 μl of the diluted amplified positive products to 44 μl of a PCR Hotstar Taq master mix (QIAGEN Ltd, Crawley, UK) and the HSV primers with the addition of M13 primer sequences [HSV-M13 forward 5'-TGTAAAACGACGGCCAGTAGCCTGTACCCCAGCAT-3'; HSV-M13 reverse 5'-CAGGAAACAGCTATGACCTGGGCCTTCACGAAGA-3']. Cycling temperatures were the same as for the HSV real-time quantitative assay, which used SYBR Green I, modified by the addition of 5 extra cycles and a final extension at 72°C for 5 min. PCR DNA product and purity were checked by electrophoresis using a 2% agarose gel. PCR products with no primer-dimers present were purified using Qiagen DNA mini-kits (QIAGEN Ltd, Crawley, UK). When primer-dimers were observed specific PCR amplicons were gel purified (QIAGEN Ltd, Crawley, UK). Purified PCR products were then sent to the Wellcome Trust Centre for Human Genetics, Oxford, UK for dye-primer Sanger sequencing on an ABI 3100 (Applied Biosystems, Inc, Warrington, UK) capillary automated sequencer.

### Detection of *Chlamydia trachomatis *pgp3 gene

The presence of *C. trachomatis *DNA was detected and quantified by Quantitect SYBR Green I on the ABI 5700 sequence detection system using *C. trachomatis *pgp3 primers [forward primer 5'-GATGCGGAAAAAGCTTACCA-3'; reverse primer 5'-TGAATAACCCGTTGCATTGA-3']. These primers amplified a product of 193 b.p. from the multicopy cryptic chlamydial plasmid. PCR cycling conditions were as recommended by the manufacturer annealing at 59°C for 30s and extension at 72°C for 20s for 40 cycles. Standards of 10^6 ^to 10 copies per reaction of *C. trachomatis *pgp3 amplicons and negative controls were included in each PCR reaction to generate a standard curve and quantities of the unknown samples estimated as before.

### Serology

Serum anti HSV-2 IgG was detected using the Kalon IgG kit (Kalon Biologicals, Ashgate, UK) and followed the manufacturer's instructions. Detection of antibodies to HIV-1 and HIV-2 in serum was done using Murex ICE HIV 1.2.0 ELISA Test kit (Murex, Dartford, Kent, UK). Reactive samples were then subjected to further testing using Monospecific ELISA, Murex ICE HIV-2 for HIV-2 diagnosis and Wellcozyme HIV Recombinant for HIV-1 (Murex, Dartford, Kent, UK). Diagnoses were confirmed on a second serum sample collected two weeks after the first sample. For Hepatitis B the Abbott Determine™ (Abbott Laboratories, Illinois, USA) HBsAg qualitative immunoassay was used to detect Hepatitis B surface Antigen (HBsAg) in serum samples by following the manufacturer's instructions. Serum samples from patients were also screened for *T. pallidum *using MACRO-VUE Rapid plasma Reagin (BD Biosciences, Oxford, UK) test kit and following manufacturer's protocol. Positive samples were confirmed using a *T. pallidum *haemagglutination assay, Micro syph TP-200 (Axis-Shield Diagnostics LTD, Huntingdon, UK).

### Microbiology

Gram stained slides were observed for the presence or absence of Lactobacilli, BV associated organisms, *Mobiluncus *and clue cells. Diagnosis of BV was based on the Nugent Score. Cervical swabs were used to make smears on slides, Gram stained and observed for Gram-negative intracellular diplococci. Culture for the isolation of *N. gonorrhoea *was performed on Thayer Martin's medium supplemented by vitox. Any positive cultures were tested for oxidase and carbohydrate oxidation as confirmation of *N. gonorrhoea*.

### *Candida, Trichomonas vaginalis *and clue cells

A few drops of saline was used to make a wet preparation of the high vaginal swab and observed under a light microscope for the presence of *Candida*, *T. vaginalis *and clue cells (granulated epithelial cells with *Gardnerella vaginalis *attached).

### Statistical analysis

HSV viral copy numbers were log transformed before statistical analysis. Statistical analysis was carried out in EPI Info, SPSS and Minitab. Kruskal-Wallis and χ^2 ^tests were used as indicated in the results.

## List of Abbreviations

CDPI_3_: tripeptide 1,2-dihydro-(3*H*)-pyrrolo [3,2-*e*]indole-7-carboxylate

Ct: Cycle threshold

CVL: Cervicovaginal lavage

DNA: Deoxyribonucleic acid

EIA: Enzyme immunoassay

ELISA: Enzyme-Linked Immunosorbent Assay.

gG: glycoprotein G

GUD: Genital Ulcer Disease

GUM: Genito-Urinary Medicine

HBsAg: Hepatitis B surface Antigen

HIV: Human Immunodeficiency Virus

HSV: Herpes Simplex Virus

Ig: Immunoglobulin

MAb: Monoclonal antibodies

MGB: Minor Groove Binder

MRC: Medical Research Council

PBS: Phosphate Buffered Saline

PCR: Polymerase Chain Reaction

qPCR: quantitative Polymerase Chain Reaction

SNP: Single Nucleotide Polymorphism

STD: Sexually Transmitted Disease

STI: Sexually Transmitted Infection

T_m _Melting temperature

## Competing interests

The author(s) declare that they have no competing interests.

## Authors' contributions

The study was designed by MJH and EANA; experimental work was done by EANA, MJH and AN; interpretation and laboratory work was conducted by MJH, EANA, AN, SK and RB; EANA, RLB, SK and MJH were responsible for analysis of results and preparation of the manuscript.
